# Complete Genome Sequence of a Foot-and-Mouth Disease Virus of Serotype A Isolated from Vietnam in 2013

**DOI:** 10.1128/genomeA.00948-15

**Published:** 2015-08-20

**Authors:** Ji-Hyeon Hwang, Tho Dang Nguyen, Duoung Thuy Mai, Su-Mi Kim, Jong-Hyeon Park, Byounghan Kim, Thanh Long To, Kwang-Nyeong Lee

**Affiliations:** a Foot and Mouth Disease Division, Animal and Plant Quarantine Agency, Anyang, Gyeonggi, Republic of Korea; b National Center for Veterinary Diagnostics, Department of Animal Health, Hanoi, Vietnam

## Abstract

The complete genome sequence of a foot-and-mouth disease virus (FMDV) found in an isolate collected in northern Vietnam in 2013 appears to be closely related to a genetic cluster formed with isolates from China, Mongolia, and Russia in 2013. All of these are classified to fall within the Sea-97 lineage, for which little complete genome data are available.

## GENOME ANNOUNCEMENT

Foot-and-mouth disease virus (FMDV) belongs to the genus *Aphthovirus* in the family *Picornaviridae* and causes a highly contagious vesicular disease in cloven-hoofed animal species. There are seven serotypes (Euroasiatic serotypes A, O, C, and Asia1, and South African territories [SAT] serotypes SAT1, SAT2, SAT3), among which no cross-serotype protection is expected (1). Foot-and-mouth disease (FMD) is endemic in southeast Asia, and recently some lineages of serotypes O and A, indigenous to the region, have spread to China, South Korea, Mongolia, and Russia (2, 3).

In this study, we have isolated a virus of serotype A in the saliva collected from a cow (female, 7 years old) in Bac Ninh province, Vietnam in 2013. cDNA of the viral RNA was constructed using a mixture of random primers and oligo(dT)-primer with Superscript III (Invitrogen, USA). Based on the sequence of virus A/VN/03/2009 (GenBank accession no. GQ406249), 10 pairs of nucleotide primers were designed to cover the whole genome and used successfully, while the 5′ terminal noncoding genomic region was determined using the rapid amplification system of cDNA ends (Invitrogen, USA). All the sequencing reads were assembled using the DNAStar program (version 5.1; DNAStar, Inc., Madison, WI, USA) into a single contig, which is 8,229 bp long and encodes 2,332 amino acids through a single reading frame.

This isolate was most closely related to a virus collected in Guangdong, China, in 2013 (A/GDMM/CHA/2013, GenBank accession no. KF450974) with identities on the VP1 protein coding region for nucleotide and amino acid sequences of 96.00% (608/636 bp) and 94.84% (202/213 amino acids), respectively. Both of these viruses are classified within the Sea-97 lineage and appear to be in the same category with genotype IX, within the Asia topotype of serotype A. Because this lineage has caused outbreaks recently in China, Mongolia, Russia, Thailand, Vietnam, and Taiwan (subclinical cases), the understanding of its comparative genetic diversity and antigenic relationship to the available vaccine strains is very necessary. However, there are very limited genomic sequences, even partial, of this lineage in the public database. Recently, prior to this study, complete genomic sequences of this lineage have been reported for only six Vietnamese isolates collected in 2009 (4) and a South Korean isolate collected in 2010 (5).

The complete genomic sequence of the isolate from Vietnam (2013) reported in this study will serve as a reference for further study of the Sea-97 lineage, which is particularly critical in Asia because of the diversification and regional spread.

### Nucleotide sequence accession number. 

This complete genomic sequence of A/VN/T11D/2013 has been deposited in GenBank under the accession no. KJ608371.
